# Secondary syphilis masquerading as stasis dermatitis of the lower extremities

**DOI:** 10.1016/j.jdcr.2026.06.058

**Published:** 2026-07-03

**Authors:** Claudia N. Dubocq-Ortiz, Camilla A. Cascardo, Yolanda R. Helfrich

**Affiliations:** a University of Puerto Rico School of Medicine, San Juan, Puerto Rico; b Department of Dermatology, University of Michigan Medical School, Ann Arbor, Michigan

**Keywords:** secondary syphilis, syphilis, venous stasis dermatitis

## Case Description

A 59-year-old man with no significant past medical history presented with a 3-week history of a mildly pruritic eruption on the bilateral lower extremities. The eruption began as discrete erythematous papules that he attributed to poison ivy exposure (Fig 1) and progressed to diffuse bilateral lower extremity erythema, warmth, and edema despite treatment with topical triamcinolone 0.1% ointment.

**Fig 1:**
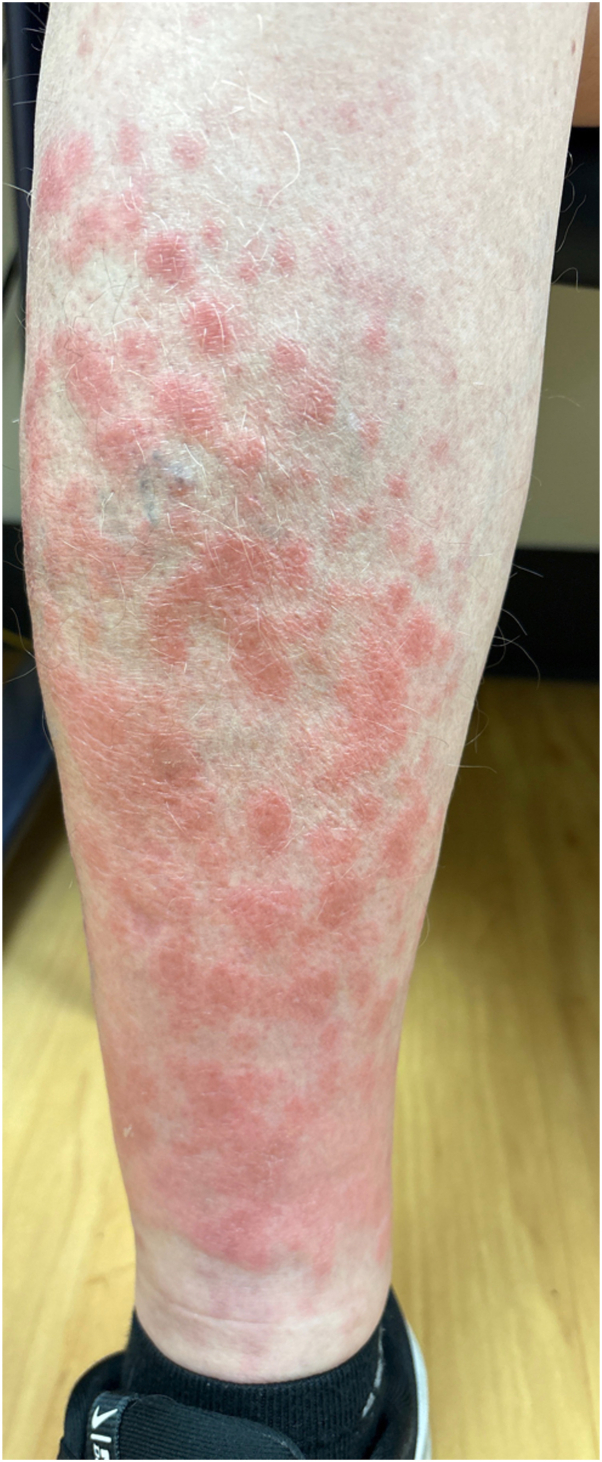
Discrete erythematous, mildly scaly pruritic papules coalescing on the lower extremity.

Review of systems was notable for fatigue, worsening arthralgias involving the knees and ankles, and mild upper respiratory symptoms. He denied recent medication changes, travel, immobilization, chest pain, dyspnea, or genitourinary symptoms.

Physical examination demonstrated erythematous papules with collarettes of scale coalescing into thin plaques on the distal lower extremities with bilateral 2+ pitting edema and tortuous veins (Fig 2, *A* and *B*). No lesions were identified on the palms, soles, oral mucosa, genitalia, trunk, or upper extremities. A punch biopsy was obtained for histopathologic evaluation.

**Fig 2:**
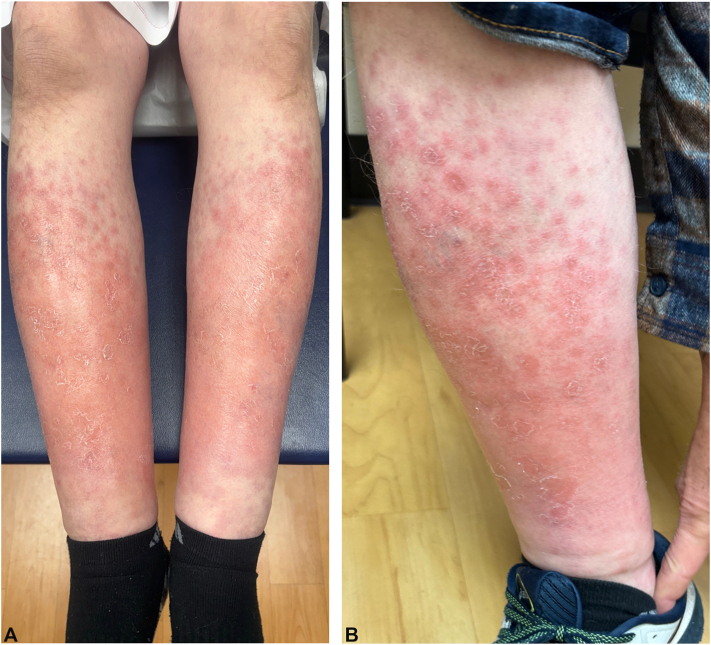
**A-B**, Erythematous papules with collarettes of scale coalescing into thin plaques associated with bilateral pitting edema.

**Question: Which diagnosis best explains this presentation?**

**A.** Acute allergic contact dermatitis

**B.** Early lipodermatosclerosis

**C.** Secondary syphilis

**D.** Pigmented purpuric dermatosis

**E.** Histiocytoid Sweet syndrome

## Answer and Discussion

C. Secondary syphilis

Secondary syphilis classically presents as a symmetric papulosquamous eruption involving the trunk, extremities, palms, and soles.^1^ However, atypical presentations may mimic inflammatory or vascular dermatoses.^2^

The eruption was initially favored to represent acute stasis dermatitis, with allergic contact dermatitis and histiocytoid Sweet syndrome also considered. However, the degree of bilateral edema was disproportionate in a patient without known venous insufficiency, prompting further evaluation for an unusual cause, such as hypoalbuminemia. Review of laboratory studies obtained by the patient’s primary care physician revealed a recently reactive rapid plasma reagin titer of 1:128. Subsequent history revealed intercourse with men and inconsistent condom use. Additional studies demonstrated positive antinuclear antibody, elevated erythrocyte sedimentation rate, and elevated C-reactive protein, while serum albumin, creatinine, and urinalysis were within normal limits.

Histopathologic examination showed epidermal acanthosis with a lichenoid, plasma cell-rich infiltrate. Treponemal immunostaining highlighted numerous spirochetes within the epidermis and superficial dermis (Fig 3), confirming secondary syphilis.

**Fig 3:**
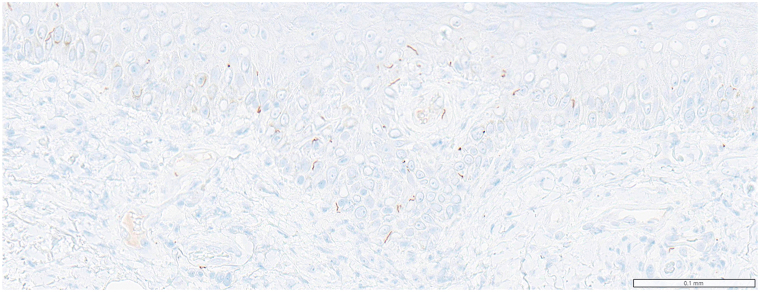
Spirochete immunostaining highlighting numerous *Treponema pallidum* organisms within the epidermis and superficial dermis.

Lower extremity edema in secondary syphilis is uncommon and is most often reported in association with syphilis-related nephropathy or nephrotic syndrome.^3^ In contrast, this patient had normal renal function, albumin, and urinalysis findings. Another distinctive feature was localization of the eruption to the bilateral lower extremities with sparing of classic sites of involvement. Previous reports have described atypical lower extremity presentations, including plantar condyloma lata and ulcerative papules and plaques mimicking pyoderma gangrenosum.^4^^,^^5^ This case further expands the spectrum of atypical presentations by demonstrating secondary syphilis masquerading as stasis dermatitis.

Clinicians should maintain suspicion for syphilis in patients with epidemiologic risk factors, even when lesions are localized and mimic more common inflammatory dermatoses. Serologic testing and skin biopsy remain essential to avoid diagnostic delay and ongoing transmission.

### Declaration of generative AI and AI-assisted technologies in the writing process

During the preparation of this work, the authors used AI for grammar and formatting edits. After using this tool, the authors reviewed and edited the content as needed and take full responsibility for the content of the publication.

## Conflicts of interest

None disclosed.
